# Oxidative Stress–Related Genome‐Wide Mendelian Randomization Identifies Causal Genes for Coronary Artery Disease

**DOI:** 10.1155/cdr/4594769

**Published:** 2026-03-24

**Authors:** Hongliang Zhang, Jingsheng Feng, Wence Shi, Guannan Niu, Yanqin Zheng, Zhenyan Zhao, Moyang Wang, Zheng Zhou, Zhe Li, Yongjian Wu

**Affiliations:** ^1^ Coronary Heart Disease Center, Fuwai Hospital, Chinese Academy of Medical Sciences and Peking Union Medical College, National Center for Cardiovascular Diseases, Beijing, China, fuwaihospital.org; ^2^ Department of Cardiology, The Third Hospital of Bazhou City, Bazhou City, Hebei Province, China

**Keywords:** causal inference, colocalization, coronary artery diseases, Mendelian randomization, oxidative stress

## Abstract

Although oxidative stress (OS) links to the pathogenesis of coronary artery disease (CAD), its underlying genetic mechanisms remain unclear. Through summary data–based Mendelian randomization (SMR) and colocalization, this research seeks to assess the potential causal links between OS‐related genes and CAD. Summary‐level data on the methylation, expression, and protein abundance levels of OS‐related genes were obtained from the corresponding quantitative trait loci (QTL) studies. We obtained genome‐wide association study summary statistics for CAD from a previous study (discovery), the FinnGen and UK Biobank (replication). Two‐sample MR analysis was conducted to verify the associations between key genes expressions and meta‐analysis cohort of CAD risk. Mediation analysis was conducted to evaluate the mediating role of gene expression variation in the causal pathway linking methylation levels of key loci to the risk or progression of the disease. We identified 35 methylation loci, 7 genes, and 12 proteins in the discovery cohort. By integrating multiomic data, we identified *SMARCA4*, *NAGLU, SREBF1*, *RPTOR,* and *HLA-B* as potential causal targets associated with CAD. The two‐sample MR analysis once again confirmed that the expressions of the *SMARCA4, SREBF1,* and *HLA-B* genes in the meta‐analysis cohort showed a significant association with the risk of CAD, and this association was consistent with the direction of the SMR. Furthermore, both the expression and methylation of *SMARCA4* were positively associated with CAD, and the direct and indirect effects of SMARCA4 methylation were confirmed. In summary, our results identified potential causal associations between *SMARCA4*, *NAGLU, SREBF1*, *RPTOR, HLA-B*, and CAD. The findings highlight the necessity for further exploration into the underlying etiology of CAD.

## 1. Introduction

As one of the leading causes of morbidity and mortality worldwide, coronary artery disease (CAD) is a prevalent cardiovascular condition characterized by the obstruction of coronary arteries, often due to atherosclerotic plaques [1, 2]. CAD typically manifests with symptoms such as angina, myocardial infarction, or sudden cardiac death [3]. Despite the availability of treatment options, the causes of CAD remain multifactorial and not fully understood, with high‐risk factors such as hypertension, diabetes, hyperlipidemia, smoking, obesity, alcohol consumption, and genetic disposition [2].

Oxidative stress (OS) occurs when an overabundance of reactive oxygen or nitrogen species exceeds the cellular antioxidant capacity, leading to potential biomolecular damage [4]. OS is a key pathological mechanism contributing to the initiation and progression of CAD through its detrimental effects on lipids, vascular cells, myocardium, and antioxidant defenses [5, 6]. For instance, excessive reactive oxygen species (ROS) perturb redox signaling in vascular cells, leading to endothelial dysfunction, compromised vasodilation, enhanced vascular smooth muscle cell proliferation and migration, and the advancement of atherosclerosis [7]. Thus, understanding the roles of OS is crucial for CAD research. Many experimental and observational studies have explored the causal relationship between OS and CAD by manipulating the expression of OS‐related genes. However, study outcomes are often conflicting, mostly due to inconsistent methodologies that fail to account for confounding effects, thereby obscuring causality. Therefore, a comprehensive analysis of OS‐related genes in CAD using a robust method is necessary to determine whether OS is a cause or consequence of CAD.

Mendelian randomization (MR) offers an alternative to conduct causality assumptions that cannot be readily obtained from conventional observational studies [8]. By utilizing randomly allocated genetic variants as instrumental variables (IVs), MR investigates the causal connections between two factors, thereby mitigating confounding bias and reverse causality [9, 10]. Summary data–based Mendelian randomization (SMR) utilizes independent genome‐wide association study (GWAS) summary statistics and quantitative trait locus (QTL) data to identify causal genes from GWAS results [11]. Using this approach, potential causal associations between OS‐related genes and CAD were identified, followed by a heterogeneity in dependent instruments (HEIDI) test [12]. Here, an SMR analysis was executed to investigate the potential associations of OS‐related genes′ methylation, expression, and protein abundance with the risk of CAD.

## 2. Materials and Methods

### 2.1. Study Design

**Figure 1:**
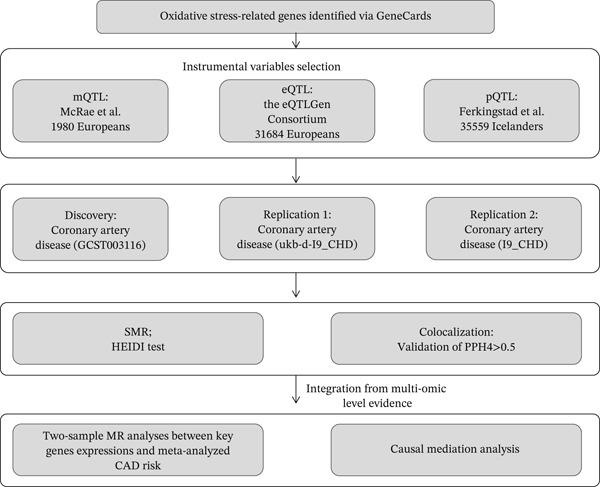
Overall study design of the MR analysis. A flow chart depicts how the SMR analysis was conducted in this study.

Numerous biological studies have demonstrated that genetic variants influencing gene expression result in varying expression levels among individuals with different genotypes [13]. Furthermore, when a gene impacts a phenotype, the phenotype manifests differently across genotypes [14]. This phenomenon aligns closely with the principles of MR [15]. However, the current availability of phenotype, single‐nucleotide polymorphisms (SNPs), and gene expression data is insufficient to adequately perform MR analysis. To address this limitation, we employ the SMR method, which integrates summary‐level data from independent GWAS with methylation expression quantitative trait loci (mQTL), expression quantitative trait loci (eQTL), and protein quantitative trait loci (pQTL) study data. This method facilitates the identification of genes whose expression levels are linked to disease phenotypes [16]. Figure 1 summarized the overall study design.

### 2.2. Data Sources

GWAS summary statistics for CAD were obtained from publicly available databases. The primary discovery dataset [17], which comprised 60,801 cases and 23,504 controls of European ancestry, was not only large‐scale but also meticulously curated to ensure a high degree of accuracy in its findings. The use of UK Biobank [18] and the FinnGen [19] datasets for validation purposes further underscores the necessity of this data selection. These additional datasets provide external replication and confirmation of the initial findings, enhancing the credibility and generalizability of the results. The detailed information for each phenotypic outcome data was provided in Table S1.

One thousand, one hundred and forty‐three OS‐related genes were extracted from the GeneCards database (v5.20, https://www.genecards.org) using the keyword “oxidative stress” and selecting those with a relevance score ≥ 7 [20]. QTLs can uncover the relationships between SNPs and variations in DNA methylation, gene expression, and protein abundance. Blood mQTL summary data were generated from a meta‐analysis of two European cohorts: the Brisbane Systems Genetics Study (*n* = 614) and the Lothian Birth Cohorts (*n* = 1366) [12]. Blood eQTL summary statistics were obtained from eQTLGen, encompassing genetic data of blood gene expression in 31,684 individuals from 37 datasets [21]. Data on genetic associations with circulating protein levels were sourced from a pQTL investigation conducted by Ferkingstad et al. involving 35,559 individuals from Iceland [22].

### 2.3. SMR Analysis

The detailed information on the SMR method has been described previously [11]. In brief, SMR integrates GWAS, mQTL, eQTL, and pQTL summary statistics to explore the association between methylation loci, gene expression, protein abundance, and the incidence of CAD. Leveraging top associated cis‐QTLs, SMR achieved enhanced statistical power compared with conventional MR analysis, particularly in scenarios with large sample sizes and independent datasets for exposure and outcome. Cis‐QTLs were selected based on a ± 1000 kb window around the gene of interest and a significance threshold of 5.0 × 10^−8^ [23]. SNPs with allele frequency differences exceeding 0.2 between datasets were excluded. Thresholds for pQTL, mQTL, and eQTL were set at 0.05. To differentiate between pleiotropy and linkage, we employed the HEIDI test, with *p* − HEIDI < 0.05 indicating potential pleiotropy and leading to exclusion from the analysis. False discovery rate (FDR) adjustments were performed using the Benjamini–Hochberg method. Associations meeting the criteria of an FDR‐corrected *p* value < 0.05 and *p* − HEIDI > 0.05 were considered for colocalization analysis in mQTL and eQTL datasets. Similarly, associations meeting a nominal *p* value < 0.05 and *p* − HEIDI > 0.05 were analyzed for pQTL datasets. The different thresholding strategies were based on the distinct biological roles of these QTLs. mQTL and eQTL, representing upstream regulatory events, required a stricter threshold (FDR‐corrected*p* < 0.05) to filter out noise and identify robust signals. In contrast, pQTL, reflecting downstream functional effectors closer to disease phenotypes, used a more lenient threshold (nominal*p* < 0.05) to avoid missing true biological signals.

### 2.4. Colocalization Analysis

Colocalization analysis is aimed at determining whether two independent association signals at the same genetic locus could be attributed to a shared causal variant. Specifically, in our study, we investigated whether the mQTL, eQTLs, pQTL, and the incidence of CAD shared a common variant in a particular region, using a Bayesian localization approach [24]. The R package “coloc” was used to perform the analysis. In these analyses, five different posterior probabilities are reported, corresponding to the following hypotheses: H0 (no causal variants for either trait), H1 (a causal variant for gene expression only), H2 (a causal variant for disease risk only), H3 (distinct causal variants for two traits), and H4 (the same shared causal variant for both traits) [25]. When GWAS signals and QTLs are found to colocalize, it suggests that the GWAS locus may influence the complex trait or disease phenotype by modulating gene expression or splicing [26, 27]. For colocalization analysis, all SNPs within 1000 kb upstream and downstream of each top cis‐QTL were retrieved to determine the posterior probability of H4 (PPH4) [28]. The colocalization analysis was conducted with the P21 parameter set at 5 × 10^−5^, and subsequent results from this analysis were filtered using a PPH4 value greater than 0.5, indicating strong evidence of colocalization between GWAS and QTL associations [29].

### 2.5. Integration Analysis of Multiomic Evidence

The integrative analysis is aimed at delineating the causal associations within the complex interplay of mQTL, eQTL, pQTL, and CAD, as well as the interplay among QTLs. With the methylation loci in the mQTL‐GWAS results as the exposure and the expressions of these genes in the eQTL‐GWAS results as the outcome, SMR was conducted to clarify the relationship between mQTL and eQTL, with the thresholds set as above. Similarly, SMR analysis was performed with the expression of genes in eQTL‐GWAS as the exposure and protein abundance in the pQTL‐GWAS results as the outcome. The integration of multiomic results was to identify key genes that may have a causal effect on CAD and to pinpoint the molecular mechanisms linking genetic variation to CAD, providing a more detailed understanding of the disease′s etiology.

### 2.6. A Two‐Sample MR Analysis

To enhance the robustness of the relationships between OS‐related genes and CAD, we meta‐analyzed the CAD datasets from GCST003116, FinnGen, and UK Biobank. The meta‐CAD dataset was used as the outcome dataset for the subsequent two‐sample MR analysis. For the exposure, SNPs significantly associated with the expression levels of eQTLs at a genome‐wide level were screened with a threshold of *p* < 1 × 10^−5^. Then SNPs with a minor allele frequency (MAF) > 0.01 were selected. To reduce redundancy, linkage disequilibrium (LD) among SNPs was excluded according to R2 < 0.3 within a 500 kb window. Furthermore, *F*‐statistics for these IVs were calculated using the formula F = R2∗(N − 2)/(1 − R2), of which *R*2 represents the proportion of phenotypic variance explained by a single SNP, and *N* refers to sample sizes [30]. SNPs exhibiting an *F*‐statistic less than 10 were considered to be poor IVs and were therefore excluded [30].

A two‐sample MR analysis was executed between the eQTLs and meta‐analyzed CAD risk. We applied inverse variance weighting (IVW) [31], weighted median [32], weighted mode [33], and MR‐Egger [34] to calculate the odds ratio and confidence interval, with IVW as the main approach. The findings were presented through scatter plots showcasing IV impacts on exposures and outcomes and forest plots which illustrate SNP effect estimates.

### 2.7. Sensitivity Analysis

To exclude pleiotropy, MR‐Egger was utilized, where pleiotropy was indicated if the intercept term is significant (*p* < 0.05) [35]. Cochran′s *Q* was utilized for heterogeneity identification among the IVs, with *p* value over 0.05 dictating no heterogeneity [36]. Furthermore, MR‐PRESSO and Radial MR were applied for outlier elimination (*p* < 0.05) and correcting for horizontal pleiotropy [35]. Steiger tests were employed to determine the causal directions [37]. A leave‐one‐out approach was applied in the MR analysis, where each SNP was sequentially omitted to evaluate its individual impact on the overall causal inference. Funnel plots were also generated to showcase the publication biases.

### 2.8. Mediation Analysis

We employed a two‐step approach to explore potential mediation effects. The Sobel method was utilized to assess the indirect effect, which was calculated as *β*a × *β*b [38]. The standard error of the indirect effect was computed using the medci function from the “RMediation” package [39]. Both the total effect and the mediation effect were significant, and the mediation proportion was positive.

### 2.9. Statistical Analysis

All statistical analyses were performed using R (v4.3.0). The R package “CMplot” was used for Manhattan plot generation, and “forestplot” for forest plot generation. The code for SMRLocusPlot and SMREffectPlot was sourced from Zhu et al. [11].

## 3. Results

### 3.1. OS‐Related Gene Methylation and CAD

Results for potential causal effects of OS‐related mQTL on CAD are visualized in Figure 2a,b (Table S2). A total of 35 methylation loci (19 genes) passed multiple testing correction. Of the identified signals, 31 near 15 unique genes were found to have strong colocalization evidence support (PPH4 > 0.5), including *SMARCA4* (cg12411068). Specifically, *SMARCA4* methylation at cg12411068 (OR = 1.269, 95% CI = 1.136–1.417) and *HLA-B* methylation at cg27139419 (OR = 1.077, 95% CI = 1.033–1.123) were linked to an increased CAD risk. Moreover, *RPTOR* methylation at cg06872548 (OR = 0.948, 95% CI = 0.92–0.977) and cg09592546 (OR = 1.032, 95% CI = 1.015–1.05) showed diverse associations with CAD risks. Similarly, *SREBF1* exhibited diverse associations with CAD, with cg01538166, cg03845363, cg08129017, cg08455676, cg09796270, cg16460860, and cg26588076 positively associated with CAD and cg15030378 showing the opposite. Among these identified CpG sites, the association for cg19186356 (*ALDH2*), cg13175981 (*MCL1*), cg19475224 (*NOS3*), and cg02459519 (*PECAM1*) were replicated in the FinnGen cohort (Table 1), whereas these associations were not replicated in the UK Biobank. The detailed associations in FinnGen and the UK Biobank were provided in Tables S3 and S4.

Figure 2SMR analyses of the potential causal effects of OS‐related gene mQTL on CAD. (a) Forest plot depicting the association between gene methylation and CAD and (b) Manhattan plot for associations between gene methylation and CAD.

**Figure:**
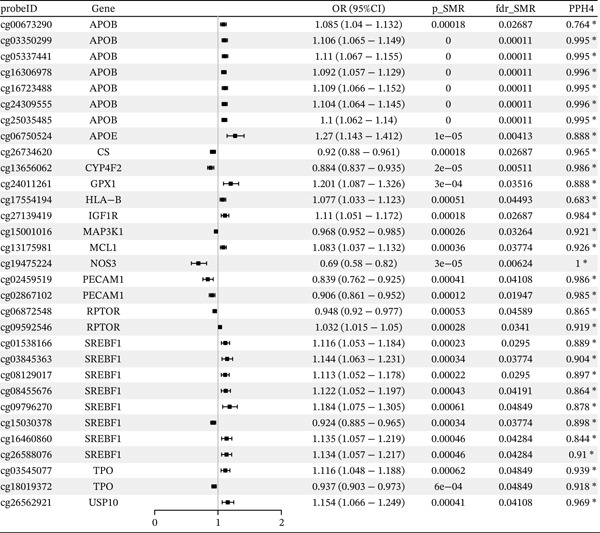
(a)

**Figure:**
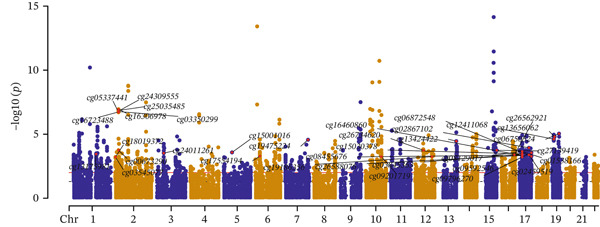
(b)

**Table 1:** Causal effects of the identified gene methylation on CAD in FinnGen cohort.

Probe ID	Top SNP	Symbol	*F* *D* *R*_*S* *M* *R*	OR_*S* *M* *R*	95% CI_SMR
cg19186356	rs10744773	ALDH2	0.009038537	1.15	1.071–1.235
cg13175981	rs11204679	MCL1	0.014118687	1.072	1.033–1.113
cg19475224	rs3918226	NOS3	0.001758332	0.756	0.667–0.857
cg02459519	rs2070784	PECAM1	0.004661374	0.836	0.767–0.912

### 3.2. OS‐Related Gene Expression and CAD

Potential causal effects of OS‐related gene expression on CAD are presented in Figure 3a,b (Table S5). After multiple testing correction, a total of seven genes were found to be associated with CAD (FDR < 0.05 and *p* − HEIDI > 0.05), in which *SMARCA4* (OR = 1.375, 95% CI = 1216–1.555, FDR = 0.0003) and *RPTOR* (OR = 1.139, 95% CI = 1.058–1.227, FDR = 0.0348) were positively associated with an increased risk for CAD, and the rest exhibited the opposite: *SREBF1* (OR = 0.893, 95% CI = 0.844–0.946, FDR = 0.0140), *NAGLU* (OR = 0.792, 95% CI = 0.71–0.884, FDR = 0.0069), *PTPN11* (OR = 0.784, 95% CI = 0.686–0.896, FDR = 0.0308), *PPIA* (OR = 0.874, 95% CI = 0.808–0.945, FDR = 0.0441), and *HLA-B* (OR = 0.934, 95% CI = 0.9–0.971, FDR = 0.0313) were negatively associated with an increased risk for CAD (Table 2). Of the identified signals, five genes were found to be colocalized with CAD incidence (PPH4 > 0.5). None of these genes were replicated in the UK Biobank and FinnGen (Tables S6 and S7).

Figure 3SMR analyses of the potential causal effects of OS‐related gene eQTL on CAD. (a) Forest plot depicting the association between gene expressions and CAD and (b) Manhattan plot for associations between gene expression and CAD.

**Figure:**
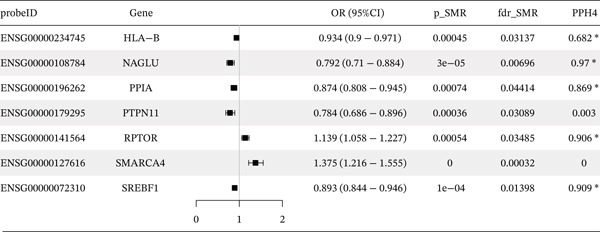
(a)

**Figure:**
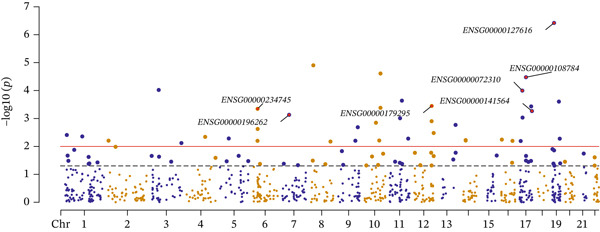
(b)

**Table 2:** Causal effects of the identified gene expression on CAD in GCST003116.

Symbol	*F* *D* *R*_*S* *M* *R*	OR_*S* *M* *R*	95% CI_SMR
SREBF1	0.013983328	0.893	0.844–0.946
NAGLU	0.006962848	0.792	0.71–0.884
SMARCA4	0.000316204	1.375	1.216–1.555
RPTOR	0.034847575	1.139	1.058–1.227
PTPN11	0.03088928	0.784	0.686–0.896
PPIA	0.044139549	0.874	0.808–0.945
HLA‐B	0.031371715	0.934	0.9–0.971

### 3.3. OS‐Related Protein Abundance and CAD

Potential causal effects of OS‐related protein abundance on CAD are presented in Figure 4a,b (Table S8). In total, 12 proteins were found to be associated with CAD at the nominally significant level (*p* value < 0.05 and *p* − HEIDI > 0.05), in which PGD (OR = 1.296, 95% CI = 1.005–1.67, *p* = 0.046), IL1RN (OR = 1.123, 95% CI = 1.007–1.254, *p* = 0.038), ANGPT1 (OR = 1.268, 95% CI = 1.063–1.512, *p* = 0.008), F2 (OR = 1.243, 95% CI = 1.047–1.476, *p* = 0.013), and NAGLU (OR = 1.043, 95% CI = 1.005–1.083, *p* = 0.026) were positively associated with an increased risk for CAD, and the rest exhibited the opposite: GPX7 (OR = 0.964, 95% CI = 0.932–0.997, *p* = 0.035), ACP1 (OR = 0.982, 95% CI = 0.964–0.999, *p* = 0.038), PXDN (OR = 0.859, 95% CI = 0.746–0.988, *p* = 0.034), VEGFA (OR = 0.936, 95% CI = 0.879–0.997, *p* = 0.039), PLAU (OR = 0.861, 95% CI = 0.797–0.93, *p* = 0.0001), JUND (OR = 0.579, 95% CI = 0.367–0.915, *p* = 0.019), and COMT (OR = 0.73, 95% CI = 0.579–0.92, *p* = 0.008). After multiple testing correction, only PLAU (OR = 0.861, 95% CI = 0.797–0.93, *p* = 0.021) exhibited significant association with CAD (FDR < 0.05 and *p* − HEIDI > 0.05) (Table 3). In the replication cohort of FinnGen, the associations between PGD (OR = 1.298, 95% CI = 1.089–1.548, *p* = 0.004), PXDN (OR = 0.888, 95% CI = 0.794–994, *p* = 0.004), F2 (OR = 1.202, 95% CI = 1.046–1.382, *p* = 0.009) protein abundance, and CAD were consistent with those of the discovery cohort (Table 4). Similarly, the associations between PXDN (OR = 0.994, 95% CI = 0.988–1, *p* = 0.039), VEGFA (OR = 0.997, 95% CI = 0.994–0.999, *p* = 0.017), ANGPT1 (OR = 1.013, 95% CI = 1.005–1.02, *p* = 0.001), JUND (OR = 0.981, 95% CI = 0.965–0.997, *p* = 0.021), and CAD were also replicated in the UK Biobank (Table 5). The detailed associations in FinnGen and the UK Biobank were provided in Tables S9 and S10.

Figure 4SMR analyses of the potential causal effects of OS‐related protein abundance on CAD. (a). Forest plot depicting the association between protein abundance and CAD and (b) Manhattan plot for associations between protein abundance and CAD.

**Figure:**
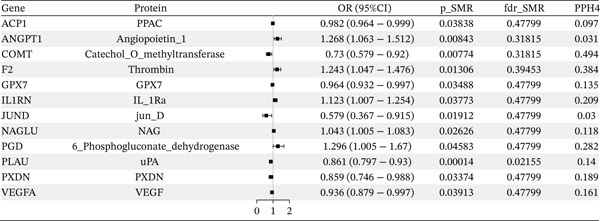
(a)

**Figure:**
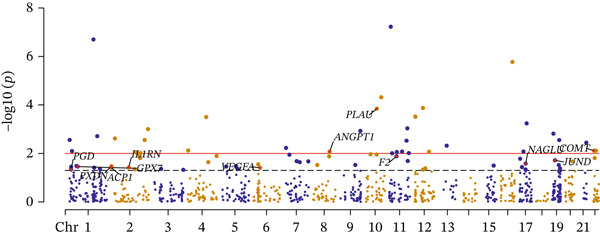
(b)

**Table 3:** Causal effects of the identified protein abundance on CAD in GCST003116.

Probe ID	*p*_*S* *M* *R*	*F* *D* *R*_*S* *M* *R*	OR_*S* *M* *R*	95% CI_SMR
PGD	0.04583378	0.477987279	1.296	1.005–1.67
GPX7	0.03487581	0.477987279	0.964	0.932–0.997
ACP1	0.0383827	0.477987279	0.982	0.964–0.999
PXDN	0.03374118	0.477987279	0.859	0.746–0.988
IL1RN	0.03773209	0.477987279	1.123	1.007–1.254
VEGFA	0.03912574	0.477987279	0.936	0.879–0.997
ANGPT1	0.008427868	0.318152017	1.268	1.063–1.512
PLAU	0.000142722	0.021550947	0.861	0.797–0.93
F2	0.01306376	0.394525552	1.243	1.047–1.476
NAGLU	0.02625969	0.477987279	1.043	1.005–1.083
JUND	0.0191238	0.477987279	0.579	0.367–0.915
COMT	0.007736968	0.318152017	0.73	0.579–0.92

**Table 4:** Causal effects of the identified protein abundance on CAD in FinnGen cohort.

Probe ID	*p*_*S* *M* *R*	*F* *D* *R*_*S* *M* *R*	OR_*S* *M* *R*	95% CI_SMR
PGD	0.003581309	0.110304317	1.298	1.089–1.548
PXDN	0.03952811	0.217404605	0.888	0.794–0.994
F2	0.00934859	0.141481083	1.202	1.046–1.382

**Table 5:** Causal effects of the identified protein abundance on CAD in the UK Biobank.

Probe ID	*p*_*S* *M* *R*	*F* *D* *R*_*S* *M* *R*	OR_*S* *M* *R*	95% CI_SMR
PXDN	0.03902312	0.445573678	0.994	0.988–1
VEGFA	0.01677628	0.3710328	0.997	0.994–0.999
ANGPT1	0.000903123	0.069992009	1.013	1.005–1.02
JUND	0.02154384	0.3710328	0.981	0.965–0.997

### 3.4. Multiomic Data Integration

Integrating the results of SMR analysis based on mQTLs, eQTLs, and pQTLs, there were four common signals (*SREBF1*, *SMARCA4*, *RPTOR,* and *HLA-B*) in the mQTL‐GWAS and eQTL‐GWAS results, and one signal (NAGLU) in the eQTL‐GWAS and pQTL‐GWAS results. By integrating blood mQTL and eQTL data, we performed SMR with the methylation loci of these genes as the exposure and their expressions as the outcome. At a stringent criteria (FDR SMR < 0.05 and *p* − HEIDI > 0.05), four methylation and two genes were causally associated, with cg12411068 methylation level (OR = 1.942, 95% CI = 1.577–2.393) positively correlated with *SMARCA4* expression, and methylation levels at cg03845363, cg08455676, and cg26588076 were associated with a reduced expression of *SREBF1* (Table 6). The detailed integrated associations were provided in Table S11. Since the *SMARCA4* gene and its corresponding methylation site, cg12411068, were found to colocalize with CAD risk, we suggest that increased methylation levels at cg12411068 may upregulate *SMARCA4* gene expression, thereby contributing to an increased risk of CAD. To visualize the results of our SMR analysis, we created locus plots for *SMARCA4* methylation and expression (Figure 5a,b). Furthermore, we also provided the effect plots confirming the effects between *SMARCA4* methylation and expression and CAD (Figure 6a,b).

**Table 6:** Causal effects of the oxidative stress‐related gene methylation on gene expression.

Expo_ID	Outco_gene	Symbol	*p*_*S* *M* *R*	*F* *D* *R*_*S* *M* *R*	OR_*S* *M* *R*	95% CI_SMR
cg12411068	ENSG00000127616	SMARCA4	4.29E‐10	4.29E‐10	1.942	1.577–2.393
cg03845363	ENSG00000072310	SREBF1	4.82E‐17	5.79E‐17	0.317	0.242–0.414
cg08455676	ENSG00000072310	SREBF1	1.25E‐21	2.15E‐21	0.348	0.28–0.432
cg26588076	ENSG00000072310	SREBF1	1.04E‐18	1.38E‐18	0.341	0.269–0.433

Figure 5Locus plots showing (a) *SMARCA4* and (b) *NAGLU*, their locations within the chromosome, and the negative log of the *p* values instrumental in deeming this locus significant in the SMR analysis.

**Figure:**
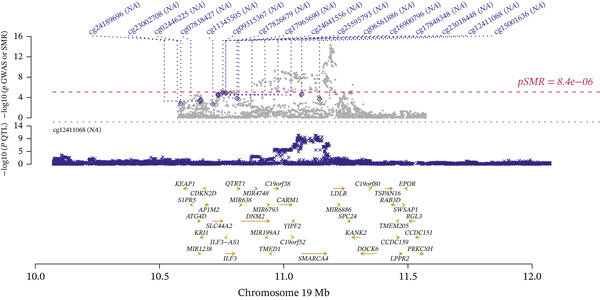
(a)

**Figure:**
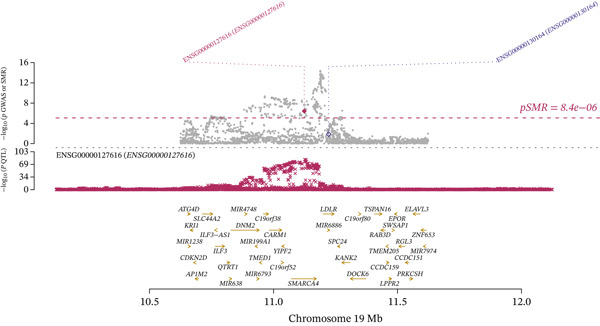
(b)

Figure 6SMR effect plots for (a) *SMARCA4* and (b) *NAGLU* and their associations with CAD.

**Figure:**
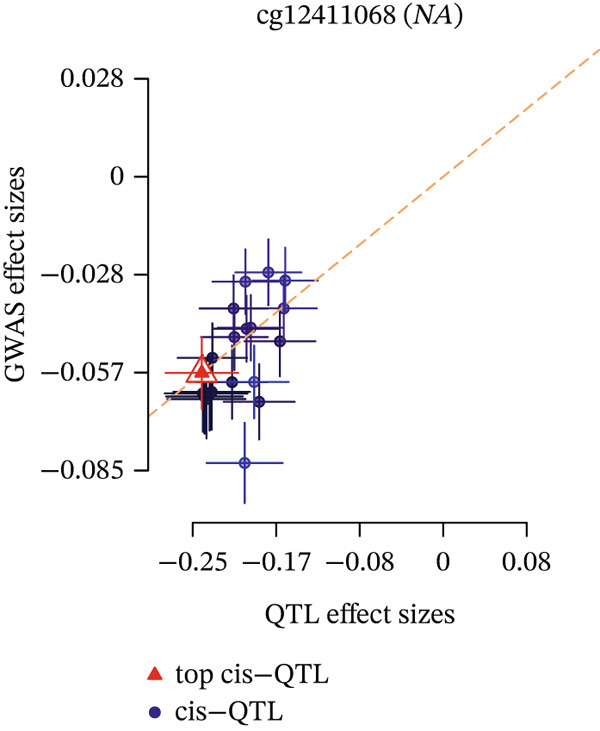
(a)

**Figure:**
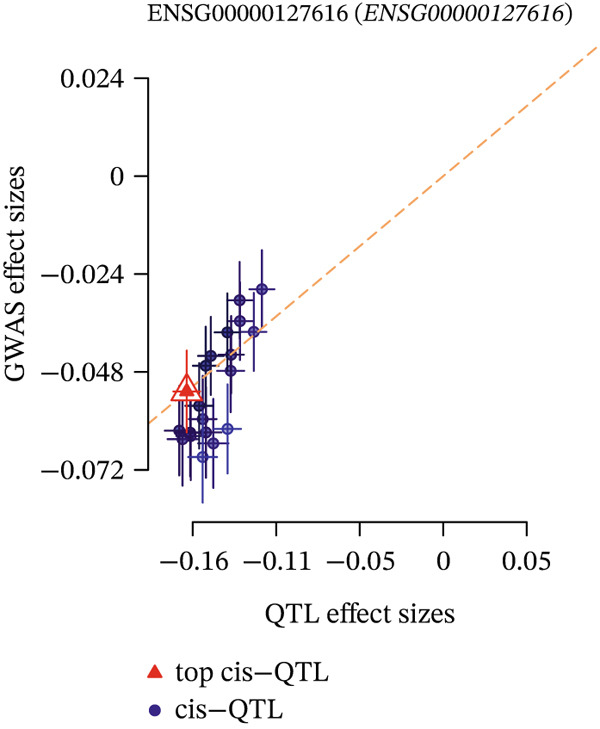
(b)

### 3.5. A Two‐Sample MR Analysis Was Conducted by Integrating the eQTL and the Meta‐GWAS Dataset of Coronary Heart Disease

To mitigate the inconsistencies across different CAD datasets, we meta‐analyzed the three CAD datasets used in this study, and explored the potential causal associations between the eQTLs of the key genes and the meta‐analyzed CAD risk. We acquired a total of 203 IVs associated with *SMARCA4*, *NAGLU*, *SREBF1*, *RPTOR*, and *HLA-B*, the *F*‐statistics of which all exceeded 10 (Table S12). MR‐PRESSO and Radial MR identified outliers for the association between expression levels of *RPTOR*, *HLA-B*, and *SMARCA4* and meta‐analyzed CAD risk (Table S13). After the removal of these outliers, MR analysis revealed a positive association between *SMARCA4* expression and meta‐analyzed CAD risk, whereas negative associations between *HLA-B* and *SREBF1* expressions and meta‐analyzed CAD risk were observed (Figure 7a and Table S13). Through MR Steiger tests, we confirmed the consistency in causal directions of these genes on CAD risk.

Figure 7A two‐sample MR analysis of gene expression and CAD risk with causal mediation of gene methylation impact. (a) The forest plot depicted the association between key gene expressions and meta‐analyzed CAD risk. OR, odds ratio; CI, confidence interval. (b) The forest plot illustrated the indirect effects of gene methylation (cg12411068, cg03845363, cg08455676, and cg26588076) on CAD through gene expression (*SMARCA4* and *SREBF1*).

**Figure:**
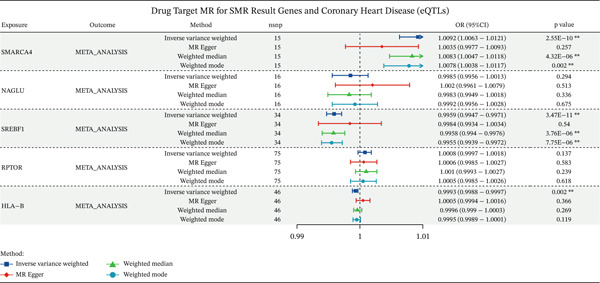
(a)

**Figure:**
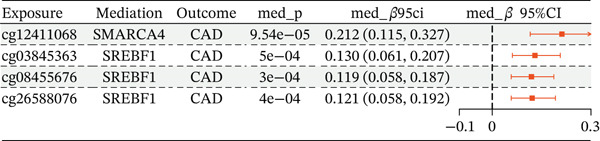
(b)

### 3.6. The Mediating Role of *SMARCA4* and *SREBF1* in CAD

To elucidate the indirect effects of gene methylation on CAD risk, we investigated the mediating role of gene expression in the relationship between methylation and CAD. Specifically, we selected genes that demonstrated significant associations in all three levels (mQTL, eQTL, and integration between mQTL with eQTL). The genes identified through this stringent selection process included *SREBF1*, *SMARCA4*, *RPTOR*, and *HLA-B*. The results indicated significant indirect effects of gene methylation on CAD risk through *SMARCA4* and *SREBF1*, suggesting the potential causal role of gene methylation in driving CAD progression (Figure 7b).

## 4. Discussion

According to published GWAS summary statistics of European ancestry from independent sources, we investigated the potential causal links between genetically predicted levels of OS‐related genes methylation, expression, protein abundance and CAD. Our SMR results indicated that *SMARCA4*, *NAGLU, SREBF1*, *RPTOR*, and *HLA-B* were putatively associated with CAD, and subsequent two‐sample MR analyses and mediation analyses further supported the mediating role of *SMARCA4* and *SREBF1* in CAD progression.

At the mQTL and eQTL levels, *SMARCA4* was found to be positively associated with CAD. Moreover, its expression was found to be positively correlated with CAD risk in the meta‐analyzed cohort, suggesting minimal effect of heterogeneity across distinct datasets. This was further substantiated by the indirect effect of its methylation locus cg12411068 on CAD risk, where the expression of *SMARCA4* played a significant mediating role. *SMARCA4* encodes the catalytic component of the SWI/SNF chromatin‐remodeling complex, which regulates transcription by disrupting histone–DNA interactions [40]. SMARCA4 is crucial for the activation of antioxidant genes and the repair of OS‐induced DNA damage, thereby helping to maintain cellular redox homeostasis [41]. Previous GWAS studies have discovered that *SMARCA4* SNP rs1122608 was linked to a reduced CAD prevalence due to its association with a more favorable lipid profile [42]. However, SNP rs1122608 was not found to be associated with *SMARCA4* expression levels [43]. Furthermore, a GWAS study conducted with Chinese Han populations demonstrated that specific alleles in the *SMARCA4* gene were associated with reduced CAD incidence in men, suggesting a sex‐related influence of *SMARCA4* variants on CAD susceptibility [44]. However, evidence on the association between *SMARCA4* and CAD from the mQTL and eQTL levels was conflicting, as both *SMARCA4* gene methylation and expression were positively associated with CAD. Therefore, we could conclude that *SMARCA4* was modestly linked to CAD, although the nature of this relationship requires further investigation to fully understand its implications.

*NAGLU* gene encodes the lysosomal enzyme alpha‐N‐acetylglucosaminidase and is involved in breaking down glycosaminoglycans (GAGs), specifically heparan sulfate [45]. Although NAGLU′s primary function is in the degradation of GAGs, its potential involvement in OS could be related to the modulation of inflammation, a process that can be influenced by the levels of heparan sulfate. *NAGLU* has been identified as a potential biomarker for CAD, especially in the context of myocardial injury (MI), where decreased protein expression has been observed in MI patients [46]. Reduced *NAGLU* expression may be linked to mechanisms of atheroprotection or tissue healing. However, direct evidence from observational and experimental studies on the association between *NAGLU* and CAD is lacking. In our study, genetically predicted levels of *NAGLU* expression and protein abundance exhibited opposite association direction with CAD, where *NAGLU* mRNA expression was negatively associated with CAD, whereas its protein levels were positively associated. This discrepancy may be partly attributed to the complex regulatory network from gene to disease. For example, posttranslational modifications such as phosphorylation or ubiquitination could alter the stability and function of NAGLU protein, leading to the observed positive association with CAD. Additionally, the tissue‐specific expression and function of *NAGLU*, as well as potential pleiotropic effects of genetic variants influencing *NAGLU*, might also contribute to this discrepancy. Further research is needed to clarify the role of *NAGLU* in CAD.

*SREBF1* encodes a transcription factor that plays a crucial role in lipid metabolism, particularly in the regulation of fatty acid and cholesterol synthesis. The expression levels of *SREBF1* have been found to be predictive of HDL cholesterol levels, suggesting that it may influence the progression of atherosclerosis by modulating lipid profiles [47]. In the context of CAD, its role in lipid metabolism is complex. On one hand, it can drive the production of molecules that are essential for maintaining cellular membrane integrity and function. On the other hand, dysregulation of *SREBF1* can lead to excessive lipid production, which, when combined with other factors such as inflammation and immune responses, can promote the development of CAD [48]. Moreover, research indicates that SREBF1 may be a potential therapeutic target for CAD. Modulating its activity could help regulate lipid levels and reduce the risk of plaque formation. For instance, in mice with diet‐induced insulin resistance, inhibition of *SREBF1* attenuates accelerated atherosclerosis [49]. Similarly, our SMR findings suggest negative associations between *SREBF1* methylation and its expression, its expression and CAD risk, and positive associations between SREBF1 methylation and CAD risk. Moreover, causal mediation analysis further suggested the mediating role of *SREBF1*, supporting both direct and indirect effects of *SREBF1* methylation in CAD progression. However, a deeper understanding of the molecular mechanisms by which *SREBF1* operates, and how it interacts with other cellular processes in the context of CAD, is necessary to develop effective treatments.

At the mQTL level, *RPTOR* exhibited diverse associations with the incidence of CAD, with methylation at cg06872548 positively associated and cg09592546 negatively associated, whereas at the eQTL level, *RPTOR* was consistently positively correlated with CAD incidence. *RPTOR* encodes a crucial component of the mTORC1 signaling pathway, which has been shown to influence cellular metabolism and autophagy, processes that are critical in managing OS [50]. Dysregulation of this pathway may lead to increased susceptibility to oxidative damage, contributing to various diseases, including cardiovascular conditions [50]. Research indicates that variants in the *RPTOR* gene have been associated with an increased risk of cardiovascular disease, potentially through suppression of mTOR [51]. The mTORC1 pathway, influenced by RPTOR, plays a role in endothelial cell function and proliferation, which are vital for maintaining vascular health [52]. Impaired mTORC1 signaling can lead to endothelial dysfunction, a precursor to atherosclerosis and CAD [52, 53]. Therefore, this evidence suggests the potential of *RPTOR* as a biomarker for the risk assessment of CAD, enabling the early identification of high‐risk individuals and the implementation of personalized measures.

At the mQTL and eQTL levels, *HLA-B* methylation loci at cg17554194 was positively associated with the incidence of CAD, whereas its expression was negatively associated. The *HLA-B* gene is a critical component of the human immune system, which encodes a surface receptor that could recognize peptides. On the cell surface, HLA‐B displays these peptides to immune cells, enabling the distinguishment between self and foreign antigens. Variations in the *HLA-B* gene have been associated with several health conditions, including CAD. For instance, *HLA-B* ∗ *07* gene is negatively correlated with CAD, suggesting that it may have a protective effect [54]. On the other hand, the haplotype formed by *HLA-B* ∗ *35* and *HLA-DR1* is significantly associated with severe CAD in heart transplant patients, indicating it may increase the risk of CAD [54]. However, direct evidence on the association between *HLA-B* and CAD is lacking. The exact mechanisms by which *HLA-B* regulates CAD pathogenesis remain elusive, warranting further investigation.

Given the modest link between key genes and CAD identified in our study, targeting these genes may hold potential for clinical translation in CAD management. For instance, small‐molecule inhibitors of *SMARCA4* have shown promise in preclinical studies for other diseases including cancers and vascular disorders, suggesting a similar approach could be explored for CAD [55, 56]. By modulating SMARCA4 activity, it may be possible to influence the expression of antioxidant genes and DNA repair mechanisms, which are crucial for maintaining cellular redox homeostasis and preventing OS‐induced damage associated with CAD. Further research is needed to develop and test such inhibitors specifically for CAD, and to determine their efficacy and safety in clinical settings. This could open new avenues for therapeutic strategies targeting the underlying molecular mechanisms of CAD.

This study represents the first evaluation of the associations between OS‐related genes and CAD using SMR and colocalization. The main strength of this study is its use of SMR, allowing simultaneous assessment of the associations between methylation, expression, and protein abundance of OS‐related genes and CAD in independent European populations. Additionally, colocalization approaches effectively eliminate potential bias caused by linkage disequilibrium. Additionally, GWAS datasets with large sample sizes increased the statistical power of our study. Nonetheless, some limitations have to be addressed. First, the association between *SMARCA4* and *NAGLU* and CAD was inconsistent at mQTL, eQTL, and pQTL levels. Second, caution is advised when interpreting posterior probabilities (PPH4) in colocalization analysis, as a low PPH4 does not necessarily indicate a lack of evidence for colocalization, particularly when PPH3 is also low due to insufficient statistical power.

## 5. Conclusions

Our findings indicated potential causal relationships between OS‐related gene methylation, expression, and protein abundance with CAD, highlighting the significance of SMARCA4 and SREBF1 and their upstream regulation in CAD pathogenesis. However, further studies are needed to minimize bias inherent in observational studies and to elucidate the mechanisms by which these genes regulate CAD pathogenesis and to develop therapeutic strategies to reduce disease severity and improve prognosis.

**Nomenclature**
OS: oxidative stress
CAD: coronary artery disease
SMR: summary data–based Mendelian randomization
QTL: quantitative trait loci
ROS: reactive oxygen species

## Author Contributions

(I) Conception and design: Hongliang Zhang, Jingsheng Feng, and Wence Shi; (II) administrative support: Zhe Li and Yongjian Wu; (III) provision of study materials or patients: Guannan Niu, Yanqin Zheng, and Zhenyan Zhao; (IV) collection and assembly of data: Wence Shi, Guannan Niu, and Yanqin Zheng; (V) data analysis and interpretation: Hongliang Zhang, Moyang Wang, and Zheng Zhou; (VI) manuscript writing: All authors. Hongliang Zhang and Jingsheng Feng contributed equally to this work.

## Funding

This study was supported by the CAMS Innovation Fund for Medical Science (2022‐I2M‐C&T‐B‐042), National High Level Hospital Clinical Research Funding (2022‐GSP‐QN‐2), and Chinese Cardiovascular Association‐Access Fund (2020‐CCA‐ACCESS‐075).

## Disclosure

All authors approved the final manuscript.

## Ethics Statement

The authors have nothing to report.

## Consent

The authors have nothing to report.

## Conflicts of Interest

The authors declare no conflicts of interest.

## Supporting Information

Additional supporting information can be found online in the Supporting Information section.

## Supporting information


**Supporting Information 1** Table S1: Basic characteristics of the study populations.

**Supporting Information 2** Table S2: Potential causal effects of OS‐related gene methylation on CAD.

**Supporting Information 3** Table S3: Potential causal effects of OS‐related gene methylation on CAD in the UK Biobank.

**Supporting Information 4** Table S4: Potential causal effects of OS‐related gene methylation on CAD in FinnGen.

**Supporting Information 5** Table S5: Potential causal effects of OS‐related gene expression on CAD.

**Supporting Information 6** Table S6: Potential causal effects of OS‐related gene expression on CAD in the UK Biobank.

**Supporting Information 7** Table S7: Potential causal effects of OS‐related gene expression on CAD in FinnGen.

**Supporting Information 8** Table S8: Potential causal effects of OS‐related protein abundance on CAD.

**Supporting Information 9** Table S9: Potential causal effects of OS‐related protein abundance on CAD in the UK Biobank.

**Supporting Information 10** Table S10: Potential causal effects of OS‐related protein abundance on CAD in FinnGen.

**Supporting Information 11** Table S11: mQTL and eQTL SMR analysis.

**Supporting Information 12** Table S12: The detailed information for the IVs used in the two‐sample MR analysis.

**Supporting Information 13** Table S13: The two‐sample MR analysis for the associations between key genes expressions and meta‐analyzed CAD risk.

## Data Availability

The GWAS summary statistics for CAD can be accessed via the UK Biobank, FinnGen, and GWAS Catalog under the search term of GCST003116 [17]. The QTLs data for OS‐related genes can be obtained via various study consortia and cohorts [12, 21, 22].
